# Nursing Home Social Services Directors Caring for Residents With Serious Mental Illness

**DOI:** 10.1093/geroni/igab046.446

**Published:** 2021-12-17

**Authors:** Denise Gammonley, Xiaochuan Wang, Kelsey Simons, Kevin Smith, Mercedes Bern-Klug

**Affiliations:** 1 University of Central Florida, Orlando, Florida, United States; 2 University of Rochester School of Medicine & Dentistry, Department of Psychiatry, Rochester, New York, United States; 3 University of Iowa, Iowa City, Iowa, United States

## Abstract

Psychosocial care for residents with serious mental illness (SMI) requires understanding of co-morbidities and careful attention to needs, rights, and preferences. Analyses of social services directors (SSDs) responses (n=924) to the National Nursing Home Social Service Director Survey considered perceived roles and competence to provide care stratified by the percentage of NH residents with SMI. Depression screenings and biopsychosocial assessments were common roles regardless of the percentage of residents with SMI. About one-quarter lacked confidence to train colleagues in recognizing distinctions between depression, delirium and depression (23.4% unable) or to develop care plans for residents with SMI (26% unable). A bachelor’s degree (OR=0.64, 95% CI:0.43, 0.97) or less (OR= 0.47, 95% CI:0.25, 0.89) was associated with less perceived competence in care planning compared to those with a master’s degree. SSDs reported less involvement in referrals or interventions for resident aggression in homes with a high proportion of residents with SMI.

